# Heterozygous SOD2 deletion selectively impairs SERCA function in the soleus of female mice

**DOI:** 10.14814/phy2.15285

**Published:** 2022-05-17

**Authors:** Jessica L. Braun, Holt N. Messner, Riley E. G. Cleverdon, Ryan W. Baranowski, Sophie I. Hamstra, Mia S. Geromella, Jeffrey A. Stuart, Val A. Fajardo

**Affiliations:** ^1^ Department of Kinesiology Brock University St. Catharines Ontario Canada; ^2^ Centre for Bone and Muscle Health Brock University St. Catharines Ontario Canada; ^3^ Centre for Neuroscience Brock University St. Catharines Ontario Canada; ^4^ Department of Biological Sciences Brock University St. Catharines Ontario Canada

## Abstract

The sarco(endo)plasmic reticulum Ca^2+^ ATPase (SERCA) restores intracellular Ca^2+^ ([Ca^2+^]_i_) to resting levels after muscle contraction, ultimately eliciting relaxation. SERCA pumps are highly susceptible to tyrosine (T)‐nitration, impairing their ability to take up Ca^2+^ resulting in reduced muscle function and increased [Ca^2+^]_i_ and cellular damage. The mitochondrial antioxidant enzyme, superoxide dismutase 2 (SOD2), converts superoxide radicals into less reactive H_2_O_2_. Heterozygous deletion of SOD2 (*Sod2* ^+/−^) in mice increases mitochondrial oxidative stress; however, the consequences of reduced SOD2 expression in skeletal and cardiac muscle, specifically the effect on SERCA pumps, has yet to be investigated. We obtained soleus, extensor digitorum longus (EDL), and left ventricle (LV) muscles from 6 to 7 month‐old wild‐type (WT) and *Sod2* ^+/−^ female C57BL/6J mice. Ca^2+^‐dependent SERCA activity assays were performed to assess SERCA function. Western blotting was conducted to examine the protein content of SERCA, phospholamban, and sarcolipin; and immunoprecipitation experiments were done to assess SERCA2a‐ and SERCA1a‐specific T‐nitration. Heterozygous SOD2 deletion did not alter SERCA1a or SERCA2a expression in the soleus or LV but reduced SERCA2a in the EDL compared with WT, though this was not statistically significant. Soleus muscles from *Sod2* ^+/−^ mice showed a significant reduction in SERCA’s apparent affinity for Ca^2+^ when compared to WT, corresponding with significantly elevated SERCA2a T‐nitration in the soleus. No effect was seen in the EDL or the LV. This is the first study to investigate the effects of SOD2 deficiency on muscle SERCA function and shows that it selectively impairs SERCA function in the soleus.

## 1 INTRODUCTION

Reactive oxygen species (ROS) and reactive nitrogen species (RNS) are normal cellular by‐products of metabolism and include molecules such as superoxide (O_2_ **^•^** ^−^), hydrogen peroxide (H_2_O_2_), nitric oxide (NO) and peroxynitrate (ONOO**^•^** ^−^) (Di Meo et al., 2016). While both ROS and RNS are important signaling molecules at low concentrations, at higher concentrations, they are highly damaging (Brieger et al., 2012; Pacher et al., 2007). As such, there exist several cellular antioxidant systems and molecules that work to maintain this delicate balance. The superoxide dismutase (SOD) enzyme family acts to neutralize highly reactive O_2_ **^•^** ^−^ molecules to less damaging, but longer lasting and membrane‐diffusible H_2_O_2_ molecules (Brieger et al., 2012).

The importance of these antioxidant systems become apparent in studies investigating ROS and RNS and their roles in aging and disease (Bokov et al., 2004; Kudryavtseva et al., 2016; Valko et al., 2016). Research has repeatedly shown a role of oxidative stress in aging and age‐related disorders in both human and pre‐clinical rodent models (Bokov et al., 2004; Giorgi et al., 2018; Jørgensen et al., 2014; Kudryavtseva et al., 2016; Mori et al., 1998), and it was proposed as early as the 1970s that the lifespan may depend on the regulation of oxygen utilization rates, and thus ROS production (Harman, 1972). For example, it has been well‐studied that oxidative stress is implicated in motor neuron degeneration and amyotrophic lateral sclerosis (Barber et al., 2006) and that the complete loss or mutation of the SOD1 isoform, resulting in significant oxidative stress, leads to motor neuron disease in mice (Fischer et al., 2011, 2012; Joyce et al., 2015; Qaisar et al., 2019). Furthermore, high levels of ROS and RNS appear to play complex roles in cancer development due to metabolic abnormalities (Bhardwaj & He, 2020) and life‐long reductions in the mitochondrial SOD2 enzyme were associated with increased DNA oxidative damage and cancer incidence (Van Remmen et al., 2003). Taken together, it becomes clear that excessive ROS/RNS production, or the lack of antioxidant activity, can lead to damage of DNA, proteins, and lipids, resulting in cell death and damage to the organism as a whole (Bokov et al., 2004; Giorgi et al., 2018).

One protein that is highly susceptible to ROS/RNS post‐translational modifications is the sarco(endo)plasmic reticulum Ca^2+^ ATPase (SERCA) pump (Braun, Geromella, et al., 2021; Braun et al., 2019; Tupling et al., 2007; Viner et al., 1996; Viner, Williams, et al., 1999). SERCA is responsible for the active transport of Ca^2+^ from the cytosol into the sarco(endo)plasmic reticulum (MacLennan et al., 2003; Periasamy & Huke, 2001; Periasamy & Kalyanasundaram, 2007). In muscle, there are two main isoforms: SERCA1a and SERCA2a (the fast and slow isoforms, respectively), with their functions being necessary for eliciting muscle relaxation, ensuring sufficient Ca^2+^ load for subsequent contractions, and maintaining low intracellular Ca^2+^ ([Ca^2+^]_i_) levels (Periasamy & Huke, 2001; Periasamy & Kalyanasundaram, 2007). The SERCA1a isoform is most abundant in fast‐twitch skeletal muscles which rely primarily on glycolytic metabolism for energy, whereas the SERCA2a isoform is present in slow‐twitch, oxidative, and cardiac muscle (Periasamy & Kalyanasundaram, 2007). Due to the presence of highly susceptible tyrosine residues (294 and 295) on SERCA (Tupling et al., 2007; Viner et al., 1996; Viner, Ferrington, et al., 1999; Viner, Williams, et al., 1999), high levels of ROS/RNS can result in tyrosine (T)‐nitration (Gow et al., 2004; Pacher et al., 2007) of the pump, altering its protein structure and catalytic activity (Braun, Geromella, et al., 2021; Braun et al., 2019; Qaisar et al., 2019; Viner et al., 1996), displaying as reductions in Ca^2+^ affinity and/or V_max_, both leading to increases in [Ca^2+^]_i_. This in turn can negatively impact muscle form and function, including atrophy and weakness, respectively. Indeed, oxidative stress has long been associated with muscle atrophy and weakness, resulting from impaired SERCA function (Braun, Geromella, et al., 2021; Braun et al., 2019; Lokuta et al., 2005) and increased [Ca^2+^]_i_, ultimately leading to more ROS/RNS production (Eisner et al., 2013; Qaisar et al., 2019). For example, it has been shown that elevated [Ca^2+^]_i_ will activate cytosolic NADPH oxidase enzymes and promote mitochondrial Ca^2+^ uptake, perpetuating ROS/RNS production and cellular dysfunction (Fink et al., 2017; Glancy et al., 2013; Kavanagh et al., 2000).

Previous work has shown that SERCA function is impaired in *Sod1* ^−/−^ mice and this dysfunction may contribute to the oxidative stress, mitochondrial dysfunction, and muscle weakness seen in these mice (Qaisar et al., 2019). Unlike the SOD1 isoform which is mainly localized to the cytoplasm and mitochondrial intermembrane space, the SOD2 isoform localizes to the mitochondrial matrix (Miao & St Clair, 2009) and its homozygous deletion results in embryonic lethality (Li et al., 1995). Nevertheless, heterozygous reduction of SOD2 increases oxidative stress (Kang et al., 2014; Richters et al., 2011; Van Remmen et al., 2001, 2003; Williams et al., 1998) and could potentially affect SERCA. Thus, the purpose of this study was to investigate SERCA function, SERCA protein content, and SERCA‐specific tyrosine (T)‐nitration in skeletal and cardiac muscles of SOD2 deficient (*Sod*2^+/−^) female mice. We chose to examine the soleus as a representative oxidative muscle and the extensor digitorum longus (EDL) as a representative glycolytic muscle as well as left ventricle (LV) tissue.

## 2 MATERIALS AND METHODS

### 2.1 Animals

C57BL/6J wild‐type (WT) and *Sod*2^+/−^ female mice were acquired from The Jackson Laboratory (Bar Harbor, Maine, USA) and housed in an environmentally controlled room with a standard 12:12‐h light‐dark cycle and given access to food and water *ad libitum*. Euthanasia at 6–7 months of age occurred via cervical dislocation while under isofluorane anesthetic after which the soleus (oxidative muscle), EDL (glycolytic muscle), and LVs were dissected, homogenized and stored at −80°C. Five to eight animals per group were used in each experiment.

### 2.2 SERCA activity

To measure SERCA activity in soleus, EDL, and LV muscle homogenate, an enzyme‐linked spectrophotometric assay was used, as previously described (Braun et al., 2019; Braun, Teng, et al., 2021; Duhamel et al., 1985). Briefly, muscle homogenates were added to ATPase reaction buffer (100 mM KCl, 20 mM HEPES, 10 mM NaN3, 1 mM EGTA, 10 mM MgCl2, 5 mM ATP, and 10 mM phosphoenolpyruvate, pH 7.0) and NADH disappearance was measured in duplicate at 37°C over a range of Ca^2+^ additions with and without the SERCA inhibitor cyclopiazonic acid (CPA) to provide a measure of SERCA‐specific ATP hydrolysis. CPA activity rates were subtracted from all activity rates, which were then normalized to total protein content, measured using a bicinchoninic acid (BCA) protein assay. Data were then fitted onto a sigmoidal dose‐response curve to calculate the *p*Ca_50_ which is the [Ca^2+^] required to elicit ½ V_max_. Maximal activity values were calculated from the raw data.

### 2.3 Western blotting

Western blotting was employed, as previously described (Braun, Geromella, et al., 2021; Braun et al., 2019; Braun, Teng, et al., 2021), to investigate the protein content of SOD2, SERCA1a, SERCA2a, nitrotyrosine, sarcolipin (SLN), phospholamban (PLN), and heat shock protein 70 (HSP70). All images were analyzed using Image Lab Software (BioRad) and normalized to total protein in each lane using Ponceau Stains. Protein‐specific information is presented in Table 1.

**TABLE 1:** Western blotting specific details. Details regarding the protein load, electrophoresis, transfer, and primary antibody probes are presented for each protein target from homogenate and immunoprecipitation experiments

	Protein loaded (µG)	Type of gel	Membrane	Primary antibody
SOD2	Soleus: 10 EDL: 10 LV: 10	BioRad PreCast TGX 4–15% gradient gels (#4568086)	PVDF	NB100‐1992SS, Novus
SERCA1A	Soleus: 10 EDL: 2.5 IP: 10 µL eluent	BioRad PreCast TGX 4–15% gradient gels	PVDF (homogenate) and nitrocellulose (IP)	MA3‐912, ThermoFisher Scientific
SERCA2A	Soleus: 2.5 EDL: 10 LV: 2.5 IP: 10 µL eluent	BioRad PreCast TGX 4–15% gradient gels	PVDF (homogenate) and nitrocellulose (IP)	MA3‐919, ThermoFisher Scientific
SLN	Soleus: 25	Tricine	Nitrocellulose	ABT13, Sigma Aldrich
PLN	LV: 10	BioRad PreCast TGX 4–15% gradient gels	PVDF	MA3‐922, ThermoFisher Scientific
NITROTYROSINE	IP: 10 µL eluent LV: 10	BioRad PreCast TGX 4–15% gradient gels	PVDF (homogenate) and nitrocellulose (IP)	#189542, Cayman Chemical

Note

Abbreviations: EDL, extensor digitorum longus; LV, left ventricle; PLN, phospholamban; PVDF, Polyvinylidene difluoride; SERCA1/2, sarco(endo)plasmic reticulum Ca^2+^ ATPase 1/2; SLN, sarcolipin; SOD2, superoxide dismutase 2.

### 2.4 Immunoprecipitation

Immunoprecipitation experiments were performed to determine the amount of SERCA‐specific tyrosine nitration. The protocol was previously described by (Braun et al., 2019). Western blots were then performed with the eluent as per Table 1. Nitrotyrosine content was normalized to the amount of SERCA2a/1a content of each respective eluent.

### 2.5 Statistical analyses

Results are expressed as mean ± standard error of the mean (SEM). A Student’s t‐test was used for most comparisons between genotypes. Comparisons for PLN and Hsp70 were made using a two‐way ANOVA testing the main effects of muscle type (soleus vs. cardiac muscle), genotype (WT and *Sod2* ^+/−^), and their potential interaction. Tests for normality were done using a Shapiro–Wilk test. In cases where normality was not met (EDL muscle weight, *p*Ca_50_, SERCA2 content, and SERCA2 nitrotyrosine content), a non‐parametric Mann–Whitney test was conducted to compare WT versus *Sod2* ^+/−^ mice. Statistical significance was set to *p* ≤ 0.05. Outliers were identified using the ROUT method (Q = 2%) and were removed prior to analyses. All statistical analyses were performed using GraphPad Prism 8 (GraphPad Software, Inc. Ca, USA).

## 3 RESULTS

### 3.1 Heterozygous Sod2 deletion reduces SOD2 protein content in muscles of female mice, but does not affect muscle weights

**FIGURE 1:**
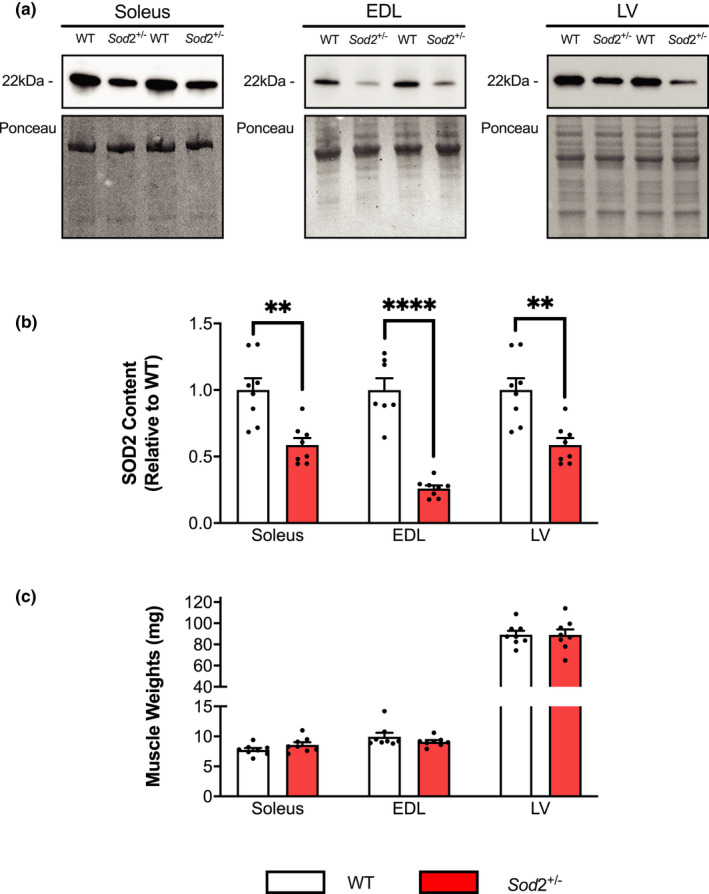
Superoxide dismutase 2 (SOD2) protein content and muscle weights of the soleus, EDL, and left ventricle (LV). (a) Representative Western blot images of SOD2 protein content in wild‐type (WT) and SOD2^+/−^ soleus, extensor digitorum longus, and LV. (b) Densitometric analysis of SOD2 protein content in the aforementioned tissues shows significant reductions in SOD2 content in SOD2^+/−^ mice compared to WT in all tissues. (c) No differences in muscle weights were observed across genotypes. SOD2 protein content values are mean ± SEM and are presented relative to WT, muscle weights are presented in mg. Data was analyzed using Student’s *t* test or Mann–Whitney test. **p < 0.01, ****p < 0.0001 (*n* = 7–8 per group)

In assessing SOD2 protein content through Western blotting in the soleus, EDL, and LV of female WT and *Sod*2^+/−^ mice (Figure 1a), significant reductions were observed in all tissues of *Sod*2^+/−^ mice compared to WT (Figure 1b). Muscle weights (in mg) were unaffected by genotype across all muscles investigated (Figure 1c).

### 3.2 Sod2^+/−^ mice show reduced SERCA Ca^2+^ affinity and selective T‐nitration of the SERCA2a isoform in the soleus

**FIGURE 2:**
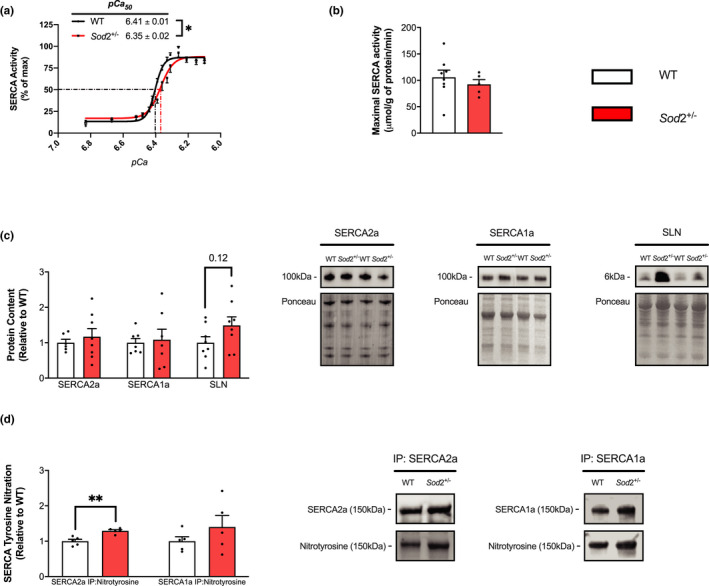
Sarco(endo)plasmic reticulum Ca^2+^ ATPase (SERCA) function is impaired and SERCA2a T‐nitration is increased in soleus muscles of SOD2^+/−^ mice. (a) SERCA activity‐*p*Ca curves in wild‐type (WT) and SOD2^+/−^ mice over Ca^2+^ concentrations ranging from *p*Ca 6.83–6.10, presented as % of V_max_. *p*Ca_50_ values are embedded in the graph with 95% confidence intervals (CIs). (b) No differences in maximal SERCA activity (μ mol/g of protein/min) were observed between genotypes. (c) Densitometric analysis and representative images of Western blots for SERCA2a, SERCA1a, and sarcolipin protein content as well as analyses and representative images of SERCA‐specific T‐nitration (d). All values are mean ± SEM and are presented relative to WT. *p < 0.05 ** *p* < 0.01, values above bars indicate *p* values using Student’s *t*‐test (*n* = 5–8 per group).

SERCA activity assays were performed in soleus muscles of WT and *Sod*2^+/−^ mice across submaximal and maximal Ca^2+^ additions (*p*Ca of 6.83–6.10). The *Sod*2^+/−^ group showed a significant rightward shift in the SERCA activity—*p*Ca curves (Figure 2a), as demonstrated with a significantly lower *p*Ca_50_ compared to WT (*p* < 0.05), but this was not accompanied by any changes in maximal SERCA activity (Figure 2b). Western blotting and densitometric analyses showed no differences in SERCA2a or SERCA1a content between genotypes, but increases in SLN content in the *Sod*2^+/−^ soleus was noted, though this was not statistically significant (*p* = 0.12, Figure 2c). Significant increases in SERCA2a‐specific T‐nitration was observed in the SOD2^+/−^ group compared to WT (*p* < 0.01), with no such effect observed with the SERCA1a isoform (*p* = 0.28, Figure 2d).

### 3.3 SOD2 deficiency does not affect SERCA function in the EDL

**FIGURE 3:**
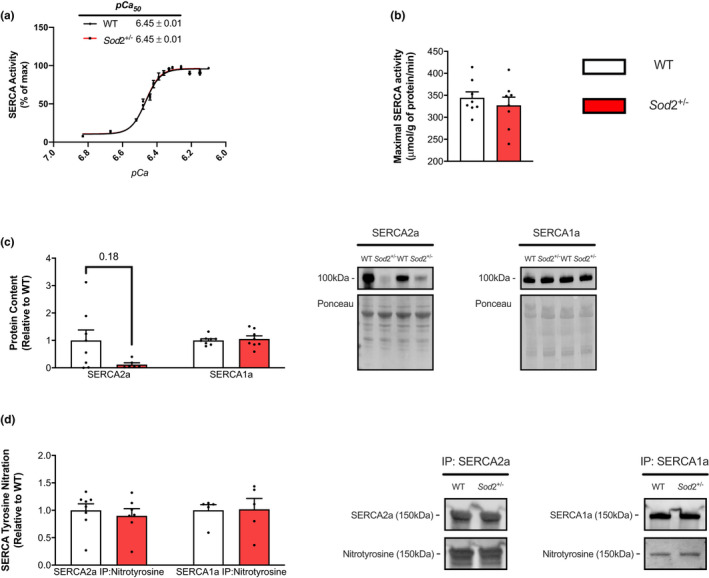
Sarco(endo)plasmic reticulum Ca^2+^ ATPase (SERCA) function is maintained in extensor digitorum longus muscles of SOD2^+/−^ mice. (a) SERCA activity‐*p*Ca curves in wild‐type (WT) and SOD2^+/−^ mice over Ca^2+^ concentrations ranging from *p*Ca 6.83–6.10, presented as % of V_max_. *p*Ca_50_ values are embedded in the graph with 95% CIs. (b) No differences in maximal SERCA activity (μ mol/g of protein/min) were observed between genotypes. (c) Densitometric analysis revealed nonsignificant reductions in SERCA2a and no differences in SERCA1a content, (d) nor in SERCA‐specific T‐nitration. All values are mean ± SEM and are presented relative to WT. Values above bars indicate *p* values using Student’s *t*‐test or Mann–Whitney test (*n* = 5–8 per group)

The SERCA activity assays in the EDL, performed across *p*Ca values of 6.83 – 6.10, show no differences between WT and *Sod*2^+/−^ genotypes for *p*Ca_50_ (Figure 3a) or maximal SERCA activity (Figure 3b). No differences were observed in SERCA1a content and while there appeared to be a reduction in SERCA2a in *Sod*2^+/−^ mice compared to WT this was not statistically significant (*p* = 0.18 with a Mann–Whitney test, Figure 3c). Neither SERCA2a nor SERCA1a showed any differences in T‐nitration between genotypes (Figure 3d).

### 3.4 SERCA Ca^2+^affinity is unaffected in the LV of Sod2^+/−^ female mice

**FIGURE 4:**
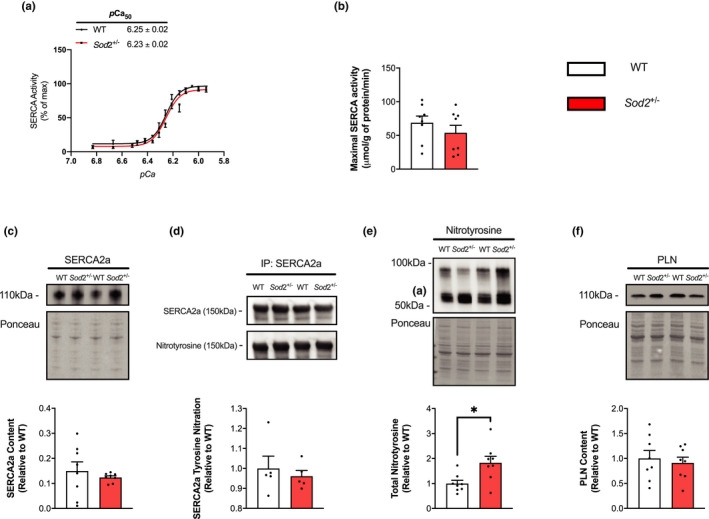
Sarco(endo)plasmic reticulum Ca^2+^ ATPase (SERCA) function is unaffected in the left ventricle of SOD2^+/−^ mice. (a) SERCA activity‐*p*Ca curves in wild‐type (WT) and SOD2^+/−^ mice over Ca^2+^ concentrations ranging from *p*Ca 6.83–5.94, presented as % of V_max_ with *p*Ca_50_ values embedded in the graph with 95% CIs. (b) No differences in maximal SERCA activity (μ mol/g of protein/min), (c) SERCA2a protein content, (d) or SERCA2a T‐nitration were observed between genotypes. (e) Total nitrotyrosine was increased in SOD2^+/−^ mice compared to WT. All values are mean ± SEM and are presented relative to WT. * *p* < 0.05 using Student’s *t*‐test (*n* = 5–8 per group)

**FIGURE 5:**
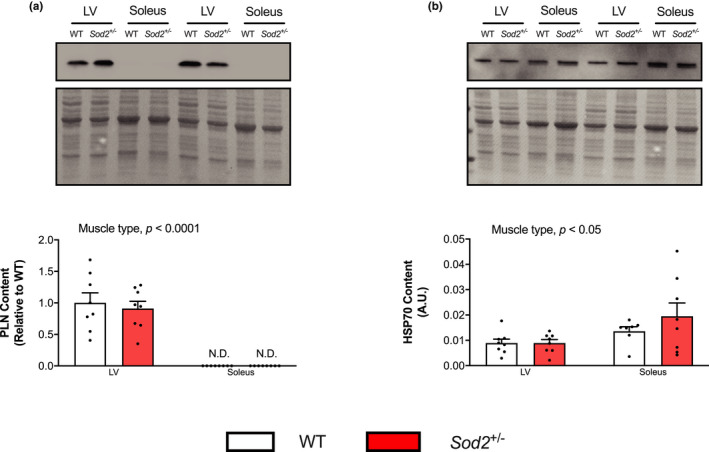
The left ventricle (LV) has more phospholamban (PLN), but less HSP70, than the soleus regardless of genotype. (a) PLN and (b) HSP70 content in the LV and soleus muscles of wild‐type (WT) and SOD2^+/−^ mice loaded on the same gel. A main effect of muscle type was detected for both PLN and HSP70 with a two‐way ANOVA. *p* values are indicated above the graph (*n *= 7–8 per group)

No differences in Ca^2+^ affinity (*p*Ca values of 6.83 – 5.94) or maximal SERCA activity was found between *Sod*2^+/−^ LV compared to WT (Figure 4a and b). SERCA2a protein content was unchanged across both genotypes (Figure 4c). While SERCA‐specific T‐nitration was unaltered between WT and *Sod*2^+/−^ mice (Figure 4d), total protein nitrotyrosine was significantly increased in *Sod*2^+/−^ LV compared to WT LV (Figure 4e, *p* < 0.05). Investigating PLN protein content between LV and soleus shows a significant main effect of muscle type (*p* < 0.0001), with no PLN being detected in the soleus when loaded on the same membrane as LV (Figure 5a). HSP70 has been shown to bind to and protect SERCA during heat stress (Fu & Tupling, 2009; Tupling et al., 2004), and we show the main effect of muscle type with significantly more HSP70 in the soleus compared to LV (*p* < 0.05, Figure 5b).

## 4 DISCUSSION

In this study, we characterized SERCA function in tissues of adult female *Sod*2^+/−^ mice to examine whether the elevated oxidative/nitrosative stress previously characterized in these mice (Kang et al., 2014; Richters et al., 2011; Van Remmen et al., 2001, 2003; Williams et al., 1998) would result in impaired SERCA function in skeletal (oxidative and glycolytic) and cardiac muscle. Our results show that with a 40–70% reduction in SOD2 protein, SERCA function in the soleus, but not EDL or LV, was affected, demonstrated by a reduction in SERCA’s apparent affinity for Ca^2+^.

The relationship between SERCA T‐nitration and impaired activity observed in the soleus muscles of this study is consistent with previous work showing an inverse relationship between SERCA2a activity and T‐nitration levels (Braun et al., 2019; Viner et al., 1996; Viner, Williams, et al., 1999). For example, SERCA Ca^2+^ uptake was shown to be reduced by 25% in rat cardiomyocytes exposed to excessive ROS (Morris & Sulakhe, 1997). Additionally, we have shown that SERCA‐specific oxidative damage correlated with reduced maximal SERCA activity in taffazin‐deficient mice, a model of Barth syndrome (Braun et al., 2019), and recent work has also demonstrated slowed muscle relaxation time in the soleus muscles of these mice (Elkes et al., 2021). Together, these studies highlight the relationship between impaired SERCA function and increased T‐nitration. However, we acknowledge that the effect of heterozygous SOD2 deletion on SERCA function reported here was mild at best given that we only observed a significant reduction in the SERCA’s apparent affinity for Ca^2+^ with no changes in maximal SERCA activity.

The reduction in Ca^2+^ affinity seen in the soleus of *Sod*2^+/−^ mice compared to WT may be partly due to the increase in SLN content, a well‐studied SERCA regulator that can reduce SERCA’s affinity for Ca^2+^ (Asahi et al., 2002; Babu et al., 2007; Rathod et al., 2021), in *Sod*2^+/−^ mice compared to WT. Despite not observing a statistically significant increase in SLN in soleus muscles from *Sod2* ^+/−^ mice, it is worth noting that SLN has been shown to play a protective role for SERCA, preventing thermal inactivation of the pump (Fu et al., 2020). Furthermore, SLN is upregulated in various models of muscle wasting including spaceflight (Braun, Geromella, et al., 2021) and muscular dystrophy (Fajardo et al., 2018; Schneider et al., 2013)—both of which also demonstrate increases in nitrotyrosine (Braun, Geromella, et al., 2021; Cleverdon et al., 2021; Gehrig et al., 2012). Thus, SLN may be upregulated in a failing effort to protect SERCA from oxidative damage, though this requires further investigation. This is consistent with previous work showing significant increases in *Sln* mRNA and protein content in both young and aged and *Sod1* ^−/−^ mice (Qaisar et al., 2018). While SERCA function was not directly measured in that study, significant increases in twitch half relaxation time, prolonged intracellular Ca^2+^ transients, and increases in calpain activity were all observed (Qaisar et al., 2018), suggestive of impaired SERCA Ca^2+^ uptake. Furthermore, recent work using a pharmacological SERCA activator, CDN1163, has demonstrated that improving SERCA function can attenuate muscle weakness and atrophy in *Sod1* ^+/−^ mice, albeit in the gastrocnemius (Qaisar et al., 2019).

In contrast with the soleus, there was no effect of SOD2 reduction in the fast‐twitch EDL muscle. This is not entirely surprising given that the soleus muscle is an oxidative muscle abundant with mitochondria, whereas the EDL is a glycolytic muscle that relies primarily on anaerobic metabolism (Schiaffino & Reggiani, 2011). Furthermore, even with consistent down‐regulation of SOD2 in both muscles, qualitative analysis shows that with equal protein loading (10 μg of protein) there is visibly less SOD2 in the EDL compared with soleus and LV. It is also important to consider differences in activation status of the muscles investigated whereby the soleus is tonically active, necessitating its oxidative nature, compared with the EDL which exhibits a more phasic activity pattern. Thus, the increased mitochondrial respiration observed with tonic muscle activation (i.e., soleus) in tandem with reductions in SOD2 may offer an explanation as to why SOD2 knockdown primarily affected the oxidative soleus. Moreover and with respect to SERCA, Viner and colleagues (Viner, Ferrington, et al., 1999) has previously shown selective nitration of the SERCA2a isoform during aging in both slow and fast‐twitch skeletal muscle sarcoplasmic reticulum (SR) vesicles, though “fast” SR vesicles showed less nitrotyrosine accumulation overall compared to “slow” SR vesicles. SERCA2a is the abundant isoform in slow‐twitch muscles such as the soleus (Periasamy & Kalyanasundaram, 2007), and the EDL is enriched with SERCA1a (Fajardo et al., 2013; Periasamy & Kalyanasundaram, 2007) providing further explanation as to why SERCA function appears to be impaired only in the soleus of *Sod*2^+/−^ mice. Indeed, we did not find any signs of SERCA1a T‐nitration in the soleus muscle from *Sod*2^+/−^ mice. Moreover, we did not find any alterations in SERCA1a or SERCA2a T‐nitration in the EDL muscles of *Sod*2^+/−^ mice, perhaps indicating that the EDL muscle is not largely affected by a reduction in SOD2 as they have relatively less protein to begin with. We did observe reductions in SERCA2a protein in the EDL of *Sod*2^+/−^ mice, however, this did not reach statistical significance nor had any negative impact on SERCA function. The reasons behind this reduction are unknown, though it could be due to potential changes in fiber type.

With the selective effect of SOD2 knockdown on the soleus muscle, we next examined the LV given that it contains ~99% SERCA2a relative to other isoforms (Lipskaia et al., 1843) and oxidative stress is the culprit of numerous cardiac diseases (reviewed in D’Oria et al., (2020)). Further, impaired SERCA function has been observed in both heart failure and dilated cardiomyopathy (Flesch et al., 1996; Frank et al., 1998; Linck et al., 1996), with two studies showing that impaired SERCA function in diseased hearts corresponded to increased T‐nitration (Braun et al., 2019; Lokuta et al., 2005). For these reasons, we hypothesized impaired SERCA function and increased T‐nitration of SERCA2a in the LV of *Sod*2^+/−^ mice compared to their WT counterparts. Interestingly, we did not observe any reductions in maximal SERCA activity or in SERCA’s affinity for Ca^2+^, unlike the soleus. Further investigation showed no changes in SERCA2a content or SERCA2a T‐nitration, though this was not due to a lack of total protein nitration. Like SLN, PLN has previously been demonstrated to have a protective effect on SERCA (Fu et al., 2020), and here we show that the LV has much more PLN content relative to soleus, which could be conferring cytotoxic protection. Notably, previous studies have shown that PLN is in fact expressed in the soleus (Braun, Geromella, et al., 2021; Fajardo et al., 2017), and we believe the lack of signal in the soleus speaks to the relative abundance of PLN in the LV compared to the soleus, which not only masks detection in the soleus but may also be preventing T‐nitration and oxidative damage to SERCA2a in the LV. We also investigated HSP70 expression in the LV versus the soleus given its known protective role on SERCA (Fu & Tupling, 2009; Tupling et al., 2004). Interestingly, we saw no changes in expression with regards to genotype, but there was significantly more HSP70 in the soleus compared to the LV. Thus, HSP70 may not explain why the LV was resistant to SERCA dysfunction in the face of increased oxidative stress in this study. Investigating the cellular mechanisms underlying the apparent protection of SERCA2a in cardiac tissue of SOD2^+/−^ mice may provide insight into possible therapeutic targets for oxidative stress‐related disorders where SERCA pumps are damaged.

Though we report only a relatively mild effect of heterozygous SOD2 deletion on SERCA, the reduction in SERCA’s apparent affinity for Ca^2+^ in the soleus could lead to Ca^2+^ disturbances at more physiological Ca^2+^ concentrations. Furthermore, increased superoxide in the mitochondrial matrix, expected in this *Sod2* ^+/−^ model, may also impair the ability of the mitochondria to sequester Ca^2+^ as previously noted with oxidative stress (Kent et al., 2021). Together, this would contribute to Ca^2+^ dysregulation and altered bioenergetics that could negatively impact contractility and muscle function of *Sod*2^+/−^ mice. However, our study is limited in that we did not assess skeletal or cardiac muscle contractility, and that we restricted our analysis to female mice. Furthermore, there are other amino acid residues found on SERCA that are susceptible to ROS/RNS (i.e., cysteine and lysine) that were not explored in this study. Nonetheless, we have found that SERCA ATPase activity is altered in the soleus muscles of SOD2 deficient mice, but not in the EDL or LV. This impairment appears to be due to increased SERCA2a‐specific T‐nitration in the soleus.

## 5 CONCLUSIONS

Our findings from this study contribute to our understanding of the *Sod*2^+/−^ mouse model and coincide with previous work demonstrating a link between SERCA function and ROS/RNS post‐translational modifications. Future studies should investigate whether the effects on SERCA function persist in male mice as well as whether protecting or improving SERCA function in the *Sod*2^+/−^ mouse model can prevent oxidative damage to the muscle.

## CONFLICT OF INTEREST

The authors declare that there are no conflicts of interest.

## ETHICS APPROVAL

All animal procedures were reviewed and approved by the Brock University Animal Care and Utilization Committee and carried out in accordance with the Canadian Council on Animal Care.

## AUTHOR CONTRIBUTIONS

JLB, HNM, JAS, and VAF conceived the study concept and design. JLB, HNM, REGC, RWB, SIH, and MSG conducted experiments for data collection. JLB, HNM, and VAF interpreted the data. JAS provided the transgenic mice. JLB and VAF wrote the manuscript that was approved by all authors.
